# Development and validation of the code of ethics for midwives in Iran

**DOI:** 10.1186/s12910-023-00963-4

**Published:** 2023-10-04

**Authors:** Masoumeh Simbar, Zahra Kiani, Soheila Nazarpour, Farah Babaei

**Affiliations:** 1 Midwifery and Reproductive Health Research Center, Department of Midwifery and Reproductive Health, School of Nursing and Midwifery, Shahid Beheshti University of Medical Sciences, Tehran, Iran; 2 Midwifery and Reproductive Health Research Center, Shahid Beheshti University of Medical Sciences, Tehran, Iran; 3 Department of Midwifery, Chalous Branch, Islamic Azad University, Chalous, Iran; 4 Department of Midwifery, Deputy of Curative Affairs, Ministry of Health, Education and Treatment, Tehran, Iran

**Keywords:** Ethics, Code of ethics, Midwifery

## Abstract

**Background:**

Considering ethical issues in midwifery care is essential for improving the quality of health services and the client's satisfaction. This study aimed to develop and validate the code of ethics for Midwives in Iran (ICEM).

**Materials and methods:**

This was a mixed sequential study that was performed in three phases including a qualitative study, a review, and the content validity assessment. The first phase was a qualitative study with a content analysis approach. The data were collected by conducting in-depth semi-structured individual interviews with 14 midwifery and ethics experts. The purposive sampling method was used to recruit the participants and sampling continued until data saturation. The data were analyzed using the conventional content analysis described by Graneheim and Lundman. Lincoln and Guba’s criteria were used to confirm the trustworthiness of the data. Then, a narrative review of the selected national and international codes of ethics for Midwives was performed to complete the items of the ICEM. For validity assessment, the face and content validity of the items of ICEM was assessed by 15 experts to calculate the content validity ratio (CVR) and index (CVI).

**Results:**

Fourteen experts were interviewed in the qualitative phase, and 207 codes were extracted from a content analysis which were categorized into 23 sub-categories and 6 main categories. The extracted codes were considered as the items for ICEM that were completed by a review of the selected national and international code of ethics for Midwives. The content validity and ratio assessment of the items demonstrated an average CVI = 0.92 and CVR = 0.85. Then, the final version of ICEM was developed with 92 items in 6 domains about; 1) "professional Commitments" with 30 items; 2) "providing midwifery services to the client and her companions” with 26 items; 3) “relationship with colleagues" with 11 items; 4) “herself” with 6 items; 5) “education and research” with 8 items; and, 6) “management” with 11 items.

**Conclusion:**

ICEM was prepared with 92 items in six sections that facilitate its use for midwives who are working in the different fields of care, counseling, education, research, and management. In this new version of the ICEM, the items related to recent social-, scientific, and technical improvements were considered for providing ethical midwifery care.

**Supplementary Information:**

The online version contains supplementary material available at 10.1186/s12910-023-00963-4.

## Introduction

Midwifery is a vital profession because midwives are responsible for maternal–fetal/neonatal care in the perinatal period, and their decisions effect maternal and infant health and the wellbeing of family members [1]. Midwives communicate with the mothers and their families during the perinatal period which is a special and critical period of human life [1]. Midwives play a significant role in the health care system by providing care and counseling during the perinatal period [2, 3]. Midwifery is a medical discipline that requires professional ethical considerations in providing care [4]. In Iran with a history of more than one hundred years of academic midwifery education, more than 30,000 midwives with Bachelor's, Master's, and Ph.D. degrees in midwifery are now working as care providers, managers, educators, or researchers in the Iranian public health system [5].

The term ‘ethics’ is a set of social rules, principles, and norms that guide people in a society, and is about right and wrong behaviors, as well as good or bad characters [6], and it involves measuring the conformity of a person's actions to a code of conduct or set of principles [7]. Medical ethics has been defined as "the analytical activity in which the concepts, assumptions, beliefs, attitudes, emotions, reasons, and arguments underlying medico-moral decision-making are examined critically" [8]. Four principles including respect for autonomy, non-maleficence, beneficence, and justice, were identified in the 1970s as guiding concepts of medical ethics [9]. Thereafter, principles such as confidentiality, privacy, consciousness, solidarity, human dignity, pluralism, tolerance, non-discrimination accountability, truth, and fidelity were also suggested by others with different cultural and historical contexts [10–12]. Then codes of ethics were developed for different medical professions gradually to help them provide care based on ethical principles [13].

Midwives also need a code of ethics to enable them to provide quality care services [14]. The code of ethics for midwives promotes self-regulation, fosters professional identity, and protects midwives and clients, as well as being used as a tool to measure professional ethical practice [7]. The American College of Nurses and Midwives (ACNM) code of ethics was first published in 1990, and then the code of ethics of the International Confederation of Midwives (ICM) was introduced in 1993. These documents and the Values Statement of the Midwifery Association of North America (MANA) guide the behavior of midwives in their practice, including providing care and counseling to women and their families, labor management, research, and health service management [15].

A code of ethics for midwives can be developed in different countries based on the country's culture and health system [16]. The code of ethics for midwives was developed in Iran in 2015 [17] with 85 items in 6 domains 1) the professional tasks (23 items), 2) providing midwifery services to the client and her companions (27 items), 3) communication with colleagues (11 items), 4) about herself (6 items), 5) in education and counseling (8 items), and 6) About management (10 items) [17]. Also, the midwifery professional practice guideline was developed based on the nursing code of ethics in 6 domains 1) altruism (6 items), 2) Honor and honesty (24 items), 3) Justice (5 items), 4) Respect (11 items), 5) Conscientiousness (19 items), and 6) Excellence (14 items) [18].

Recent studies in Iran showed an increasing expectation of improved quality of maternity services through respectful care [19], and midwives’ need for ethical knowledge to manage different situations and provide safe and proper legal and ethical maternity care [20]. Besides, Iranian midwives’ responsibilities changed after the implementation of the "Health Sector Evolution Plan" in 2015 in Iran, and midwives were introduced as a member of the “Health Care Team” and the “Family Health Care Provider” so there was some changes in the midwives’ responsibilities [21, 22]. Additionally, following the decreasing fertility rate in recent decades in Iran, promoting childbearing was announced as a critical policy, and improving the quality of maternal services was emphasized as an important strategy [23]. Also, the COVID-19 pandemic created some concerns about providing midwifery care with ethical considerations [24]. Moreover, the World Health Organization emphasized considering ethical issues in providing quality maternity care for making a positive childbirth experience [25]. Therefore, regarding several changes in midwifery responsibilities because of the increasing women's expectations about the quality of maternity services, evolution in health care system in Iran, and the related fertility and population policies, updating the midwifery ethical code seems to be necessary. Besides, the ethical code needs to be updated, evaluated, and validated periodically because of changing women's needs and the social situation [15]. Then, midwives can improve midwifery care services by providing morally and respectful services and increasing the client's satisfaction. Therefore, this study aimed to develop and validate the code of ethics for Midwives in Iran (ICEM).

## Methods

**Fig. 1:**
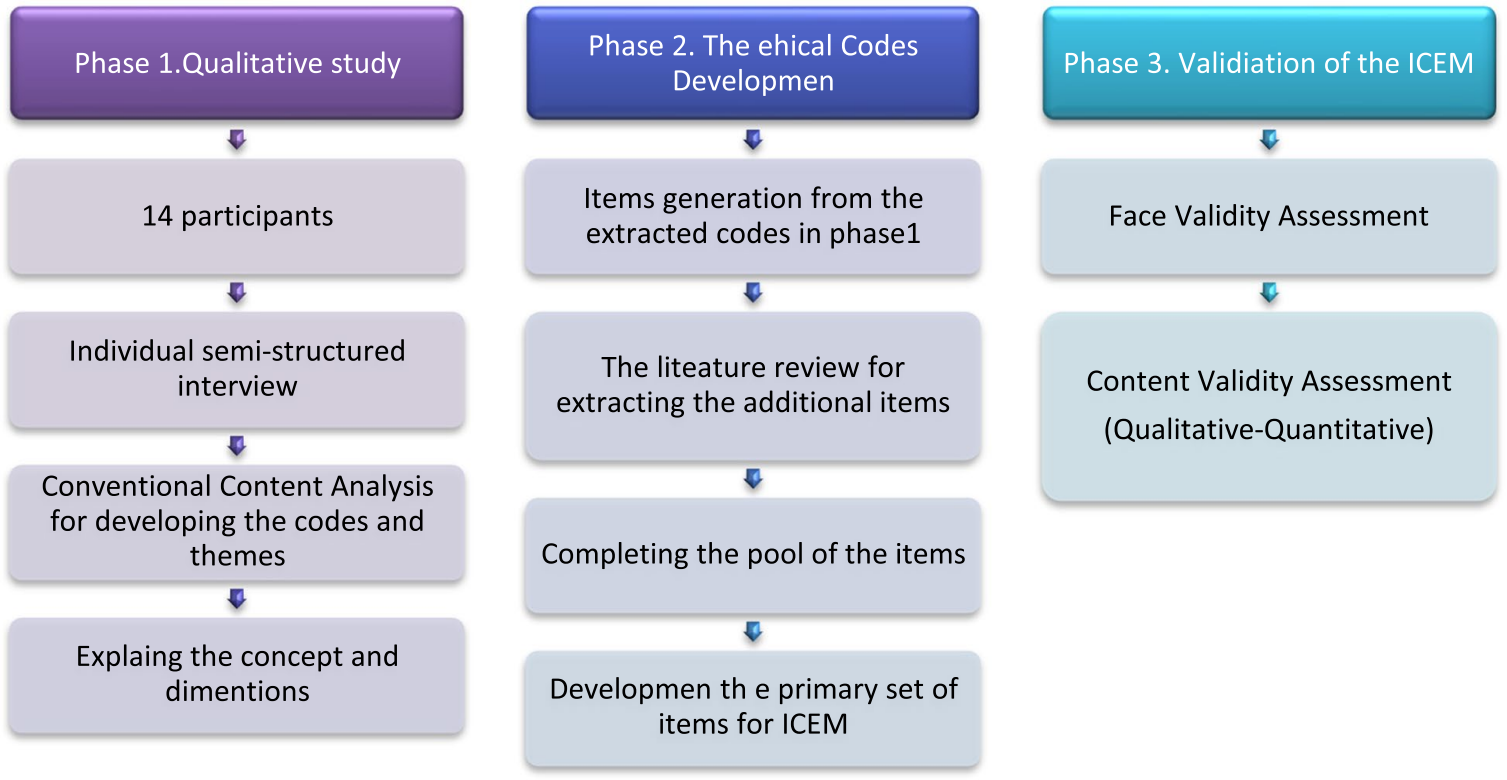
Procedure of the study to design and validate the code of ethics for midwives in Iran ICEM

This was a mixed sequential study that was performed in three phases [26] including (1) a qualitative study to develop the first set of the items for ICEM, (2) a review of the selected national and international code of ethics for Midwives to complete the items of ICEM, and then 3) validation of the items (Fig. 1).

### Phase 1) The qualitative study

The first phase was a qualitative study with a content analysis approach to generate the first set of items.

#### The design and the setting

The qualitative study aimed to explain the essential ethical considerations in different aspects of midwifery practice including midwifery care, counseling, service management, education, and research. This study was conducted in different departments that midwives provide services, including care, education and research departments in the private or public sectors in Tehran-Iran. The participants were individually interviewed in their workplaces in hospitals, health centers, or universities.

#### The participants

The participants in the qualitative part of the study were midwives who were involved in providing perinatal care services in the private and public sectors or the managers, the university faculty members of the midwifery departments, the researchers in the midwifery field, and the medical or nursing/midwifery ethics experts. They had at least two years of work experience. The participants were selected through purposive sampling from May to August 2022. Maximum variation was considered in selecting the participant's experience in care, education, research, management, and duration of work experience. The participants were individually interviewed in their offices after explaining the objectives and the process of the study and then obtaining written informed consent. Subjects’ willingness to withdraw from the study was the only exclusion criteria.

#### Data collection

The data were collected using in-depth semi-structured individual interviews with open-ended questions. The interviews began with open-ended questions such as "What is the concept of midwifery ethics" and "How midwifery ethics could be considered in midwifery services". The interviews continued to clarify the dimensions and characteristics of the subject with probing questions such as "How?", “What do you mean by that?" and “Please elaborate on this point” (Supplement 1).

#### Trustworthiness

Four criteria of credibility, confirmability, dependability, and transferability by Lincoln and Guba were used to ensure trustworthiness [27]. To increase credibility, the researcher devoted enough time to data collection. In addition, the diversity of participants by selecting midwives who worked as providers, educators, researchers, and managers as well as using different methods of data collection such as individual interviews and field notes were considered. The members, the peers, and the experts' checks of data were also used to assess the trustworthiness of the data [27].

### Data analysis

Data were analyzed using a conventional content analysis based on the criteria proposed by Graneheim and Lundman [28]. The recorded interviews were transcribed and then reviewed by the researcher several times to achieve an accurate understanding of the contents. The text was divided into meaning units, meaning units were condensed while preserving the meaning and labeled with codes. Similar codes were then categorized. The extracted codes were considered as the items of ICEM.

### Ethics approval and consent to participate

The study was approved by the ethics committee of Shahid Beheshti University of Medical Sciences, with the code “IR.SBMU.RETECH.REC.1401.113”. Before conducting the interviews, the researcher briefed the participants on the objectives and ensured the confidentiality of the information and the voluntary type of participation. All the interviews were conducted in a private and quiet room. Written informed consent was obtained from all participants.

### Phase 2) The review of the code of ethics

In this phase of the study, a review of the selected national and international code of ethics for Midwives was performed to complete the first set of the extracted items from the qualitative phase of the study. A literature search was performed in PubMed (including Medline), Web of Science, Scopus, Embase, Science Direct, and Cochrane databases as well as in the Google search platform. MeSH and keywords including ethics, code of ethics, midwifery, Midwife, and midwives were used to find documents related to the purpose of the study. No restrictions were placed on language or publication date (until May 2023).

### Phase 3) Validation of the ICEM

For the face qualitative validity assessment, 5 midwives were asked to assess the difficulty, irrelevancy, and ambiguity of the extracted items of ICEM. In the qualitative content validity assessment, 15 midwives who work as care providers, educators, researchers, and managers were asked to judge the grammar, choice of vocabulary, and placement of the items. The content validity of the items was assessed by the 15 experts in midwifery and ethics including 2 midwives working as the maternity service provider, 2 maternity service managers, 11 university faculty members, and researchers of midwifery and medical ethics. To assess the content validity ratio (CVR), the experts signified their opinions by assigning each item scores of 1 to 3, which correspond to “not essential,” “useful but not essential,” and “essential,” respectively. The scores were then calculated using the following formula: CVR = (Ne – N/2)/(N/2), where Ne is the number of experts indicating an item as “essential” and N is the total number of experts. The accepted value was determined based on Lawshe’s table and the number of experts. According to this table for 15 experts, a CVR of more than 0.49 for the item is acceptable [29]. Content Validity Index (CVI) was calculated based on Waltz and Bausell’s criteria to ensure the appropriateness of the items for measuring the content. The experts scored the relevancy of the items using the four-point Likert scale (scores 1 to 4, respectively). The CVI score of each statement was computed as the number of experts giving a rating of 3 or 4 to each item, divided by the total number of experts. Based on this index, items with a CVI higher than 0.79, between 0.70 and 0.79, and lower than 0.70 were considered suitable, needing modification, and unacceptable, respectively [30] (Fig. 1).

## Results

The findings are presented in three phases including Phase 1 the qualitative study to develop the primary items for ICEM, and then Phase 2 for completing the items of ICEM by a review of the international code of ethics for midwives, provided by the valid related organizations and other countries, and then in phase 3 the results of the validity assessment.

### Phase1: The qualitative study

Every individual interview lasted 45–60 min, and all the interviews were tape-recorded. In addition to interviews, field notes were used for data collection. Although data saturation was achieved with 12 interviews, two additional interviews were conducted for greater certainty.

The participants in the qualitative phase of the study were 14 experts, including 6 midwives who were working in the private and public sectors; 2 midwives who were maternity service managers, 4 faculty members of the university at the department of midwifery or midwifery-related research centers, and 2 experts in medical ethics (Table 1).

**Table 1:** The characteristics of participants in the qualitative phase of the study to develop midwifery code of ethics in Iran

No	Education	Field	Type of service	Location	Age (Years)	Work experience (years)	Sector
**1**	PhD^a^	Midwifery/RH^e^	Nursing/Midwifery ethics educator	University	54	26	Public
**2**	PhD	Midwifery/RH	Midwifery education and trainer	University	51	22	Public
**3**	PhD	Midwifery/RH	Midwifery education and trainer	University	47	20	Public
**4**	PhD	Midwifery/RH	Midwifery/RH researcher	University	35	7	Public
**5**	PhD	Medical Ethic	Education/Research	University	55	24	Public
**6**	PhD	Medical Ethic	Education/Research	University	45	19	Public
**7**	MSc^b^	Midwifery	Manager	Hospital	48	16	Private
**8**	MSc	Midwifery	Manager	Hospital	52	16	Public
**9**	BSc^c^	Midwifery	Care Provider	Hospital	40	14	Public
**10**	BSc	Midwifery	Care Provider	Hospital	43	17	Private
**11**	BSc	Midwifery	Care Provider	Hospital	37	10	Private
**12**	BSc	Midwifery	Care Provider	Health center	34	11	Public
**13**	BSc	Midwifery	Care Provider	Office	49	24	Private
**14**	GD^d^	Midwifery	Care Provider	Hospital	32	12	Public

^a^Doctor of Philosophy

^b^Master of Science

^c^Bachelor of Science

^d^Graduate Diploma

^e^Reproductive Health

**Fig. 2:**
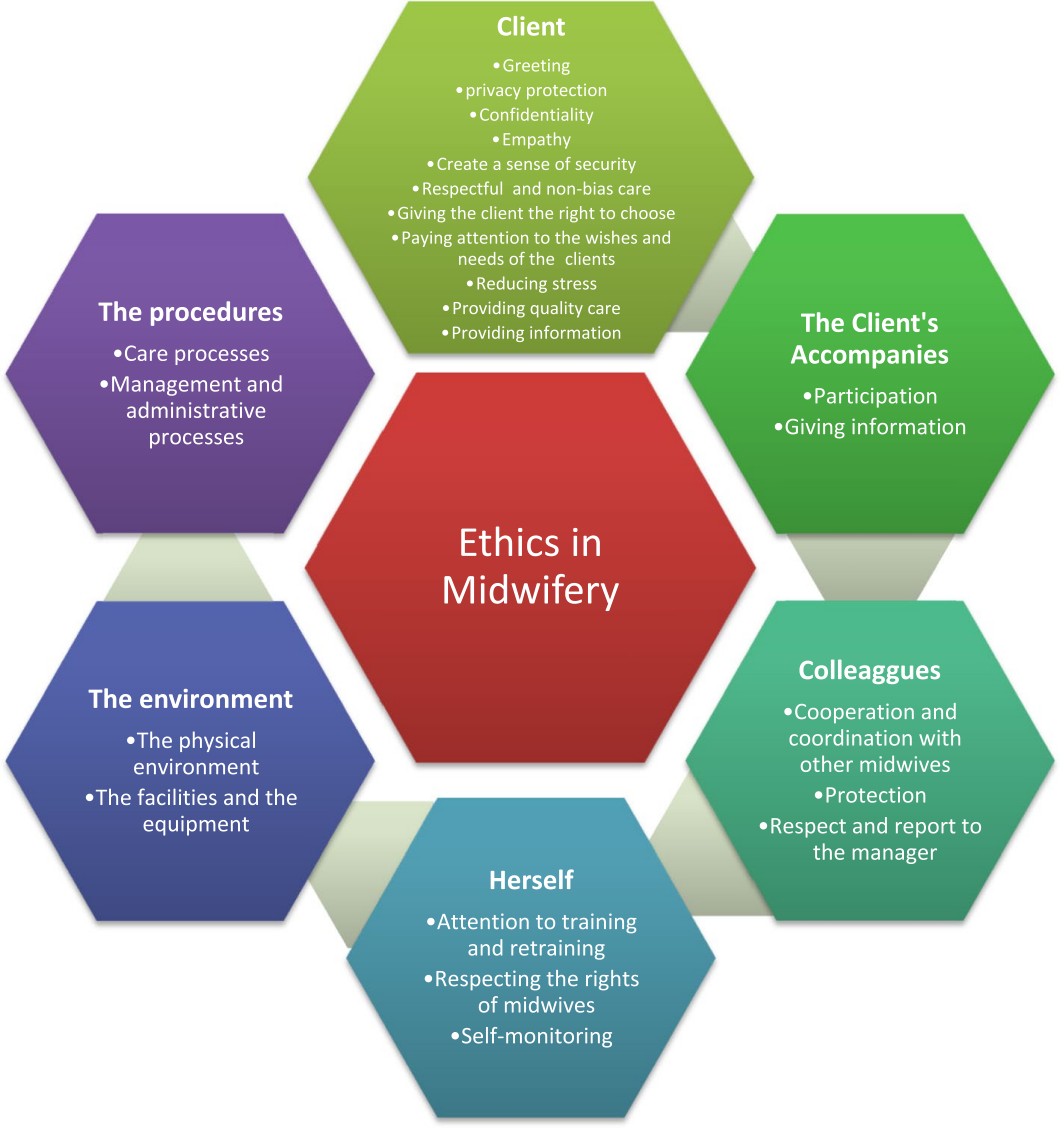
The concept and a schematic view of the concept of ethics for midwives

The findings in the qualitative phase showed the 207 codes in 23 sub-categories and 6 categories including ethical consideration about 1) the clients, 2) the clients’ companions, 3) herself, 4) the colleagues, 5) the environment, and 6) the procedures. Figure 2 shows a schematic view of the concept of ethics for midwives.

#### The midwifery ethical considerations about the clients

This category showed 84 codes in 11 subcategories including greeting, privacy, confidentiality, empathy, creating a scene of security, providing respectful and non-bias care, the right to choose, attention to the wishes and needs of clients, reducing the stress, providing quality care and providing the information (Table 2).

**Table 2:** The codes and the subcategories of the category "The midwifery ethical considerations about the clients"

Subcategory	Code
**Greeting**	Introducing and using the identification card
	Giving explanations about the rooms and care processes
	Welcoming the client on her arrival
	Making a friendly communication
	Creating a peaceful environment with a self-confident approach
	Guiding the client
**Privacy**	Privacy protection
	Considering drape for covering the client’s lap during obstetric examinations
	Providing maternity care in separate rooms
	The private counseling of the client
	Making a sense of security by considering privacy during the examination
	The right of the client for taking care of in a private environment
	Ensuring that no other people enter during the examination
**Confidentiality**	Preservation of physical and mental privacy
	The importance of confidentiality especially in the midwifery profession
	Confidentiality regarding the examination results
	Confidentiality about the client's records and information
	Protecting women's information in the electronic transfer processes
**Empathy**	The importance of empathy
	Empathy with the clients in labor and delivery rooms
	The value of empathy as the half of treatment
**Security**	Allowing the client to have companions
	Patiently interactions with clients in labor and delivery
	Making friendly and respectful communication during the examination
	Responsible for cleanliness and safety of equipment
	Be kind to the client
	Making the client feel comfortable
**Respectful and non-biased care**	Respectful care is the right of the client, especially in crowded centers
	Culturally appropriate communication
	Providing care to all clients without any cultural biases
	Providing care to everyone regardless of their illness, addiction, and problems
	Provide care without a long waiting time
	Providing respectful care regardless of the client's characteristics or problems
	Respecting the clients' Opinions
	Respecting the client's culture
	The importance of respect and dignity for the clients
	Communicate with respect and trust
	Communication with honesty and intimacy
**Decision Making**	About the normal labor and delivery
	Initiating breastfeeding before episiotomy
	To have no angiocatheter during normal labor and delivery
	Giving informed consent throughout labor and delivery
	Permitting the peripartum interventions
**The clients' needs**	Entry permission with the client's permission
	Obtaining permission for giving information to the relatives
	Providing adequate explanations based on the client's needs
	To have companion
	Considering all health dimensions
	Special needs of vulnerable clients
	In exceptional circumstances, such as disasters and covid-19 pandemic
**Reduce stress**	By giving the necessary information
	Reducing stress during labor to prevent negative effects on breastfeeding
	Patient treatment of clients for reducing stress caused by care processes
**Providing quality care**	To benefit the client
	Prevent harm
	With up-to-date scientific information
	Without discrimination
	Based on the social justice
**Providing information**	Providing information according to the understanding of the clients
	Ensuring clients' understanding of taught topics
	Giving information to the mother and answering her questions
	Avoiding confusing the patient
	Giving up-to-date information
	Using teaching aid methods
	Creating calm with proper counseling
	Providing advice to the patient to create a sense of importance
	Providing complete and up-to-date midwifery information
	Providing clear information
	Avoiding quick and unclear consultations
	Giving information about maternal rights in the midwifery care-providing system
	Using simple and sincere expressions due to the sensitivity of midwifery counseling topics
	Counseling with patience to correct a misconception
	Changing the harmful attitudes with rational reasons
	Using an interpreter for non-local language speaker clients
	Ask about the client's problems by making a friendly communication
	To be trained in prenatal care such as nutrition, exercise, and breastfeeding
	To receive explanations before and during any care process
	|To be taught about the perinatal period and care from high school
	To be informed about the advantages and disadvantages of family planning methods according to the conditions of the client's condition
	To be informed about the mechanism of the action of the family planning methods
	To be informed about the clients’ rights
	Obtaining a signature after giving sufficient information to the client
	Giving information for free and informed decision
	Giving the information about advantages and disadvantages of the new technologies
	Providers only make suggestions and are not decision-makers
	Providers give information and the clients are decision-makers

##### Greeting

Most of the participants pointed to the greeting as an important ethical point in using the identification card and welcoming, making verbal and friendly communication, and introducing the structure and processes as the client's rights. A participant said:

“We also have to go to other doctor's offices or hospitals. I like to be welcomed by the person in charge and ask about my pain” (BSc., Maternal health provider, public hospital).

##### Privacy

All the midwives participating in the study considered privacy as one of the most important ethical considerations in the midwifery profession:

"Our patients should be completely sure about their privacy. Women should be taken care of in a situation where their privacy is completely protected in terms of covering and sound." (Ph.D., the University Professor, and medical ethics researcher, university).

##### Confidentiality

All participants mentioned the necessity of confidentiality as a critical consideration, especially regarding the information obtained from the client during counseling and care.

"There are some problems where the patient has a problem but says that my family doesn't know. For example, a patient with epilepsy said My mother-in-law doesn't know, I don't want her to be told about it."(BSc., care provider, private clinic).

Some of the participants mentioned the importance of protecting women's information transfer through the internet.

"The midwife should care about the online transfer of women’s information with the name of the client, such as sending the information through the application, e-mail, fax, and social media) and consider the legal aspects for sending the information.” (MSc., Maternity service manager, Public hospital).

##### Empathy

The majority of participants pointed out the necessity of creating a sense of empathy with the client, especially during labor and delivery, and expressed its value as half the treatment:

"I always put myself in the place of the client, I treat women the way I want to be treated." (BSc., care provider, Public hospital)

##### Security

Most of the participants mentioned security as an essential right of the women in the midwifery services:

"An intimate and respectful relationship with them is necessary. We should spend time and talk to the woman and her companions in a way that they feel what we are doing for her is something that is the best for her " (BSc., care provider, Public hospital).

##### Respectful and non-biased care

Respecting and not being biased care were emphasized by the participants:

" In the midwifery practice, since women's problems are not like other medical problems such as foot pain, I believe that we should talk to the clients politely and considering their culture and literacy so that they understand and accept things well” (a midwife, researcher, a university research center).

##### Decision-making

The majority of the participants stated that the client has the right to choose or reject a treatment during the physiologic process of labor and childbirth:

"Unfortunately, the women don’t have the right to choose, they can't tell an opinion in our system. While they have the right to choose, for example, their position in the labor process” (BSc., care provider, Public hospital).

##### Considering the wishes and needs of clients

Paying attention to the client's wishes and needs was emphasized by the majority of the participants:

"The major problem is how much the client considers it the right to express her needs, and how much the care provider considers meeting the client's needs as the right " (Ph.D., the University Professor, and medical ethics researcher, University).

*“Respond to the physical, psychological, spiritual, emotional, cultural and social needs of the clients who are seeking midwifery care and also educate all clients self-care" (Ph.D., a reproductive health researcher and university professor, University”.*

"The midwife should consider the special needs of vulnerable clients such as high-risk mothers and infants, women with chronic diseases, or AIDS, or with alcohol and drug addiction, victims of violence, homeless, prisoners, refugees, and immigrants and the elderly in providing midwifery services" (MSc, a midwifery service manager, Public hospital).

“They should provide midwifery services in exceptional circumstances, such as disasters (eg. earthquake and flood) or in an epidemic or pandemic of diseases like Covid-19 pandemic.” (Ph.D., Midwifery/RH researcher, University).

##### Reduce the stress

Many participants stated that women should be aware of the labor process and the related care and services beforehand.

“I teach her in advance. For example, what position to be in, or take a deep breath to reduce her anxiety and stress?” (BSc., care provider, private hospital).

##### Providing quality care

All participants highlighted the importance of providing quality care, without any discrimination, and based on social justice to benefit the clients and prevent harm.

" The practice should result in the patient's wellness and goodness. Besides, the next principle is avoiding harm to the patient. Whatever we do, may have some complications. The next principle is social justice, which refers to the fact that care should be provided to all people, regardless of their race, gender, religion, and so on, and it should be only based on the client’s needs (Ph.D., a university professor, and medical ethics researcher, university).

"Midwives should play an effective role in promoting the health of women, family, and society" (Ph.D., a university professor and researcher in midwifery, university).

##### Providing information

All the participants mentioned the necessity of providing information as a client's right. The information should be provided properly and based on the client's needs to help them in making informed decisions.

*"First, when they come, I give some information to the client and her companion about the normal procedures of her labor and delivery and the care procedures. For clients who may not go through the normal delivery procedure, and need a cesarean section, I provide the related necessary information to both the patient and her family."(BSc., care provider, a private hospital).*

#### The ethical consideration in the relationship between midwives and the client’s companions

This category includes 15 codes in 2 subcategories including the importance of giving information to the clients and involving them in the maternal care process (Table 3).

**Table 3:** Codes, sub-categories, and categories of the ethical considerations in the midwife's relationship with companions, with herself, and with colleagues extracted from the qualitative phase of the study

Category	Sub-Category	Codes
**the relationship with the client’s companions**	**Giving information**	About the client’s labor process
		About the risks related to the client
		To make an informed decision
		For planning the waiting time
		To prevent argument
		The importance of perinatal counseling because of the special relations between mother and father
		The importance of family participation through giving information about care
		About incidents such as the sudden unwellness of the infant to prevent legal consequences
		Obtaining consent before giving information to the companions
	**Involving in the maternal care**	The importance of the family’s participation in the care procedure
		Training the companion to the involving in the care
		Let the companion be involved in the client's care during the labor
		The importance of the husband’s participation in the perinatal care
**About Herself**	**Retraining**	About scientific issues
		About ethics in midwifery
		Innovations in midwifery
		Appropriate interaction in possible conflicts
		The spirituality and spiritual approach in the midwifery care
		Ethically deal with problems with a spiritual perspective
		Ethics and the related psychology in midwifery clinical education
	**Respecting the midwives’ rights**	Monitoring of midwives' rights in the health management system
		Defend for midwifery rights and positions
		Fair division of work and shifts
		Observing the fair working hours
		Long and crowded shifts and night work prevent morality compliance
		One-to-one midwifery care in the active labor phase
		Solving the midwifery problems in the country
		Considering the difficulty of the midwifery job in deciding about working shifts
		Providing facilities such as restrooms and closets for midwives
		Clarity of job description and organizational position for effective teamwork
	**Self-monitoring and -evaluation**	Not mixing personal and work problems
		Providing conscientious services
		The need for self-sacrifice in midwifery
		Considering self-care and -safety
		Preference for the client's health
**the relationship with the colleagues**	**Cooperation**	Maximum cooperation in teamwork
		Cooperation when problems arise for shift midwives
		Counseling with team members
		Coordination in providing care
		The necessity of correct handover of patients to colleagues of the next shift
		Compromise and cooperation in difficult situations
		Timely notification and referral to the doctor
	**Support**	Respectful communication between colleagues
		Considering fair workload to reduce the stress
		To avoid making stress on colleagues
		Flexible approach to caring in teamwork
		Not talking about private issues in the presence of a client
		Avoiding non-constructive criticism
		Maintaining effective communication
		No conflict with colleagues
		Respect for colleagues' opinions and rights
		Good and friendly relationship
		Providing team care is the client's right
	**Respecting and reporting to the manager**	Attention and lessening to managers
		Considering rewards for midwives who follow the code of ethics
		Paying attention to job ranks
		A respectful relationship with managers
		Providing a complete report to the manager

##### Importance of giving information to the clients

The majority of the participants emphasized the importance of giving information to the clients about the care procedure, the client's problems, the progress, and the importance of their involvement.

“The necessary information should be given to the companion so that she/he can take care of the mother whenever it is necessary" (Ph.D., midwifery/RH researcher, a university).

##### Involving the companion in the maternal care process

All participants highlighted the need for family involvement in the care procedure and decision-making.

"Involving at least one of the companions solves many problems because they feel that a relative is also taking care of her."(MSc., midwifery service manager, a private hospital).

#### Ethical considerations regarding the midwife's relating to herself

This category includes 35 codes in 3 sub-categories including paying attention to retraining, respecting the rights, and self-monitoring and evaluation (Table 3).

##### Retraining

Retraining was mentioned as one of the most important ethical considerations about midwives themselves.

"We have been working twenty years ago. Although we have had many retraining workshops during these years on various issues, we need more. For example, we need to be trained about midwifery ethics". (BSc, care provider, a public hospital).

##### Respecting the rights

Some participants mentioned the violation of midwives' rights in the delivery room.

"When we are in the delivery room, after helping to a normal vaginal delivery or even giving an episiotomy and its repairing, and reporting the procedure in a blue sheet, the doctor would come and sign. All the trouble was for us, then, there was no name of us, and the wage for birthright is for the doctor. We do not defend our right at all.” (BSc., care provider, a public hospital).

##### Self-monitoring and evaluation

Many participants mentioned the importance of self-monitoring and evaluation in the midwifery profession.

"Midwife must evaluate herself to practice, for the patient's safety and safety of ourselves, both." (MSc., midwifery service manager, a private hospital)

#### Ethical consideration in a midwife’s relationship with the colleagues

The category includes 27 codes in 3 sub-categories including cooperation with other midwives, supporting colleagues, and respecting and reporting to the manager (Table 3).

##### Cooperation with other midwives

All participants stated that cooperation between midwives is a critical issue.

"Our colleagues should have a good and friendly cooperation for providing care and all should accept responsibility for their actions" (MSc., a maternity service manager, public hospital).

##### Supporting the colleagues

The majority of participants said that midwives should support their colleagues through respectful communication, teamwork, patience, and flexibility.

“They should avoid criticizing the colleague in front of the patient or her companion, and respect each other in the maternity services"(Ph.D., medical ethics researcher, university).

##### Respecting and reporting to the manager

All participants stressed the respectful and responsive relationship of midwives with the managers.

“One should be very respectful in dealing with the supervisor, and provide a complete report of the report her/him about patients". (BSc., midwifery care provider, a public hospital).

#### Ethical considerations in providing care and management procedures

The category includes 26 codes in 2 sub-categories including care procedures and administrative and management procedures (Table 4).

**Table 4:** Codes, sub-categories, and categories of the ethical considerations in providing midwifery Care procedures and with the work environment, extracted from the qualitative phase of the study

Category	Sub-Category	Codes
**Providing care and management Procedures**	**Care Procedures**	Safety compliance
		Making medical devices secure and sterile
		Preventing transmission of infectious diseases from the midwife to the patient or vice versa
		Paying attention to needle penetration and the use of disposable needles
		Ensuring that the mother is properly covered during childbirth
		Using sterile equipment
		Using physiologic birth processes
		Spiritual perspective in care providing
		Providing the necessary care and avoiding unnecessary invasive procedures
		Greetings, welcome, and guidance about the care process
		Being honest in writing the record
		Providing correct and complete recording
		Avoiding recording unnecessary sensitive private information
		Avoiding recording doctor's orders by phone
	**Administrative and management Procedures**	Improving standards of the services through research
		Reducing patient waiting time
		Providing follow-up services
		Monitoring for compliance with ethical regulations
		Taking an ethics exam for midwifery students’ acceptance
		Choosing students who communicate respectfully and sincerely
		Monitoring prevention tips when visiting clients with infectious diseases
		Monitoring the separation of sterile and non-sterile equipment
		Providing timely services
		Providing services with modern technology
		Supervising care provision according to perinatal care standards
		Supervising the structure of perinatal care procedures including physical environment and facilities
**Work environment**	**Environment**	Providing services in an environment with sufficient light
		Providing care in a neat and clean physical space
		Providing care in a delightful environment
		Paying attention to ventilation and sound
		Sufficient space, equipment, and facilities
		Safe work environment
		Thermally balanced environment
		Attempting to make the client feel at home
		A convenient and accessible place to provide care
		Providing care in a proper place to protect the client's privacy
		Provide the building guide
		Proper arrangement of the rooms
		Proper setting of the clinic for easy access of patients
	**Facilities and equipment**	Sterile equipment
		Facilities such as a bed for a companion
		Providing a place to rest and stretch legs
		Considering the necessary facilities for providing care in compliance with ethical principles
		Adequate facilities and equipment
		Providing cleaning facilities such as showers and cleaning training
		Adequate portable bed screen curtains to keep the clients' privacy

##### Care procedures

All participants mentioned the midwifery ethical considerations in the care procedures such as; safety compliance, making medical devices secure and sterile, preventing transmission of infectious diseases, paying attention to needle penetration and use of disposable needles, ensuring that the mother is properly covered during childbirth, using sterile equipment, using of physiologic birth processes, spiritual perspective in care providing, providing the necessary care and avoiding unnecessary invasive procedures, greeting and guiding about the care process, being honest in writing the record, providing correct and complete recording, avoiding to record unnecessary sensitive private information.

" If we have a system that has a defined and reasonable waiting time, we can make an appropriate appointment time. It is also very important for patients to make a follow-up appointment "(MSc., maternity service manager, a private hospital).

##### Administrative and management procedures

The participants also stated that ethical considerations are necessary for the administrative and management procedures and mentioned items such as; promoting the knowledge and practice of midwifery through research and development of midwifery care standards; reducing patient waiting time, providing follow-up services, monitoring for compliance with ethical regulations, taking an ethics exam for midwifery students’ acceptance, choosing students who communicate respectfully and sincerely, monitoring prevention tips when visiting clients with infectious diseases, monitoring the separation of sterile and non-sterile equipment, providing timely services, providing services with modern technology, supervising on care provision according to perinatal care standards, supervising on the structure of perinatal care procedures including physical environment and facilities.

"The midwifery service managers always should seek for the ways to promote the knowledge and practice of midwifery through research and development of midwifery care standards" (Ph.D., midwifery and reproductive health researcher and professor, University).

"Of course, our manager tries to provide a fair shift schedule with no discrimination but it is sometimes out of her control, she couldn't do it well” (BSc., maternal care provider, a public hospital).

#### Ethical considerations in the midwifery work environment

This category included 20 codes in 2 subcategories including the environment and the facilities and equipment. All participants stressed the importance of providing an appropriate work environment as well as proper equipment and facilities as the ethical consideration for providing midwifery services (Table 4).

##### Midwifery work environment

Most participants talk about the features of a proper work environment such as providing services in an environment with sufficient light, a thermally balanced environment, a neat and clean physical space, a delightful environment, with proper ventilation and sound, sufficient space, equipment and facilities, convenient and accessible place to provide care, with adequate safety, providing care in a proper place to keep the client's privacy, attempting to make the client feel at home, with proper arrangement of the rooms and appropriate setting of the clinic for easy access.

“It should have proper ventilation, there should not be too much noise so that you can concentrate on your work, there should be enough light, the right temperature” (a midwife, care provider, private hospital).

"The number of personnel should be enough, the space should be equipped, and the medical equipment should be excellent so that we can provide quality services.” (MSc., maternity service manager, a public hospital).

Table 4 shows the ethical considerations related to the care procedures and the work environment which were extracted from the interviews in the qualitative phase of the study.

### Phase 2: A review of the international midwifery code of ethics

The code of ethics provided by eight valid midwifery organizations and institutions was reviewed and summarized in Table 5.

**Table 5:** Summary of a review of the code of ethics presented in the selected organizations and countries

Country or Institute	Year	Sections	Year Summary
**International Midwifery Confederation ICM** [31]	2020	4 sections with 23 items	The purpose of this confederation is to improve the standards of midwifery care for the mother, infant, and family. The code of ethics is a guideline for midwifery education, research, and practice. Four sections including *Midwifery Relationships, Practice of Midwifery, The Professional Responsibilities of Midwives*, and *Advancement of Midwifery Knowledge and Practice* are expressed in the form of 23 items. The appearance of these items considers a woman as a person with human rights who seeks justice and equality in access to health care and emphasizes the two-way relationship of respect and trust along with the preservation of all members of society. Since The ICM is a valid midwifery institution, the midwifery code of ethics provided by this confederation was used and emphasized as the main basis for developing a code of ethics using the available texts in this study
**American College of Nursing and Midwifery ACNM** [32]	2015	3 ethical mandates and 11 ethical items	ACNM presents 3 ethical mandates to achieve the mission of midwifery to promote the health and well-being of women and newborns within their families and communities. The first mandate is directed toward the individual women and their families, the second mandate is to a broader audience for the public good for the benefit of all women and their families, and the third mandate is to the profession of midwifery to assure its integrity and in turn, its ability to fulfill the mission of midwifery. Considering these 3 ethical mandates ACNM introduced 11 ethical items including; Midwives in all aspects of professional relationships and practice will 1- respect basic human rights and the dignity of all persons; 2- respect their own self-worth, dignity, and professional integrity; 3- develop a partnership with the woman in which each shares relevant information that leads to informed decision-making, consent to an evolving plan of care, and acceptance of responsibility for the outcome of their choices; 4-Act without discrimination based on factors such as age, gender, race, ethnicity,religion, lifestyle, sexual orientation, socioeconomic status, disability, or nature of the health problem; 5-Provide an environment where privacy is protected and in which all pertinent information is shared without bias, coercion, or deception; 6-Maintain confidentiality except where disclosure is mandated by law.7-Maintain the necessary knowledge, skills, and behaviors needed for competence; 8- Protect women, their families and colleagues from harmful, unethical, and incompetent practices by taking appropriate action that may include reporting mandated by law; 9- promote, advocate for, and strive to protect the rights, health, and well-being of women, families, and communities; 10- Promote just distribution of resources and equity in access to quality health services; 11- Promote and support the education of midwifery students and peers, standards of practice, research, and policies that enhance the health of women, families, and communities
**UK Nursing and Midwifery Council UKNMC** [33]	2018	25 sections with 95 items	Treat people as individuals and uphold their dignity; 2. Listen to people and respond to their preferences and concerns; 3. Make sure that people’s physical, social and psychological needs are assessed and responded to; 4. Act in the best interests of people at all times; 5. Respect people’s right to privacy and confidentiality; 6. Always practice in line with the best available evidence; 7. Communicate clearly; 8. Work co-operatively; 9. Share your skills, knowledge, and experience for the benefit of people receiving care and your colleagues; 10. Keep clear and accurate records relevant to your practice; 11. Be accountable for your decisions to delegate tasks and duties to other people; 12. Have in place an indemnity arrangement that provides appropriate cover for any practice you take on as a nurse, midwife, or nursing associate in the United Kingdom; 13. Recognize and work within the limits of your competence; 14. Be open and candid with all service users about all aspects of care and treatment, including when any mistakes or harm have taken place; 15. Always offer help if an emergency arises in your practice setting or anywhere else; 16. Act without delay if you believe that there is a risk to patient safety or public protection; 17. Raise concerns immediately if you believe a person is vulnerable or at risk and needs extra support and protection; 18. Advise on, prescribe, supply, dispense or administer medicines within the limits of your training and competence, the law, our guidance, and other relevant policies, guidance, and regulations; 19. Be aware of, and reduce as far as possible, any potential for harm associated with your practice; 20. Uphold the reputation of your profession at all times; 21. Uphold your position as a registered nurse, midwife, or nursing associate; 22. Fulfill all registration requirements; 23. Cooperate with all investigations and audits; 24. Respond to any complaints made against you professionally; 25. Provide leadership to make sure people's well-being is protected and to improve their experiences of the health and care system
**The British Columbia College of Nurses and Midwives BCCNM** [34]	2020	4 sections with 36 items	BCCNM articulates 36 ethical items in 4 sections including 1-professional practice, with 9 items that explain the midwifery's primary responsibilities for safeguarding the well-being of the client and their newborn; 2-relationships and accountability, with 12 items about developing the midwife’s relationship of trust and partnership with the client; 3- knowledge, competency and learning with 4 items about maintaining and facilitating safe and competent midwifery practice in all environment and cultures; and 4-conduct with 11 items including midwives’ act as effective role models by maintaining both professional and ethical conduct. Midwives should not engage in any activity that would adversely affect the honor, dignity, or credibility of the profession
**Nursing and Midwifery Board Australia NMBA** [35]	2018	.4 sections with 23 items	The International items of ethics are now in effect for Australian nurses and midwives International Council of Nurses (ICN) Code of ethics for nurses and the International Confederation of Midwives (ICM) Code of ethics for midwives took effect as the guiding documents for ethical decision-making for nurses and midwives in Australia on 1 March 2018
**New Zealand College of Midwives NZCM** [36]	2023	3 sections with 28 items	The sections are 1-responsibilities to the women with 12 items; 2-responsibilities to the wider community with 5 items and 3- responsibilities to colleagues and the profession with 11 items
**Turkey Midwifery National Code of Ethics** [37]	2019	21 Items	The items refer to that a midwife should: 1-not expect personal interests and should not have any conflict of interests; 2- take the necessary precautions to protect the privacy of her patients; 3- protect honesty and reliability in her records 4- consider the unborn baby, the mother, and the newborn as separate beings; 5- prevent harm to the baby and/or the mother due to sloppy and inexperienced interventions and neglect; 6- considers the mother’s and the baby’s utmost benefits more than anything else; 7-believe that every individual has equal rights; 8-care for the breastfeeding relationship between the mother and the baby 9-never shares information about her patients with anyone, unless there is a life-threatening situation or a law that requires it; 10-care for women’s physical, emotional and spiritual values 11-improves her professional knowledge and is responsible for compliance with ethical standards and she acts in line with professional values and displays professional competence;12-protects her dignity and her professional dignity; 13-value natural birth; 14- ensure that women take advantage of all available medical resources and avoid potential hazards; 15-improve and maintain women's health and contribute to the prevention of illness; 16-fairly allocate resources and midwifery services;17-inform women about midwifery practices willingly 18-try to protect, improve and defend the rights of women and their families; 19-listen to her inner voice to judge herself; 20- improve the quality of care,21-Encoure women to make informed decisions
**Nursing and Midwifery Board of Ireland** [38]	2021	38 items in 5 sections	The Sections are: 1-Respect for the dignity of the person (16 items) 2- Professional, responsibility, and accountability)15 items) 3- Quality of practice (7 items) 4- Trust and confidentiality (7 items) 5- Collaboration with others (9 items)

The concept of ethics for midwives is considering ethics in providing the client's care, in interactions with companions, about herself, the environment, and processes. About the clients, these considerations are providing care with empathy, keeping privacy and confidentiality, as well as paying attention to the right to choose, dignity, desires, and needs, reducing stress, and providing respectful and safe quality care without any discrimination. About the companions, the considerations are giving the relevant information and permitting them to participate in the care. Cooperation, coordination, respect, and proper reporting are also necessary for colleagues. Paying attention to retraining, self-evaluation, and awareness about midwifery rights should also be considered about herself. Committed behavior regarding the maintenance and improvement of the physical environment, equipment, facilities and paying attention to the standards in care and management is necessary (Fig. 2).

### Development of the midwifery code of ethics in Iran

After extracting the items from the qualitative phase and completing the items after reviewing the international midwifery code of ethics, the items were assessed in terms of the content validity index (CVI) and ratio (CVR) by 15 experts including 2 midwives working as the maternity service provider, 2 maternity service manager, 11 university faculty members and researchers in midwifery. Then, the items were revised and edited by the research team.

Finally, the ICEM was developed by 92 items in 6 sections including:

1) "Professional Commitments" with 30 codes; 2) "Providing midwifery services to the patient and her companions” with 26 items; 3) “relationship with colleagues" with 11 items; 4) “herself” with 6 items; 5) “education and research” with 8 items; and, 6) “management” with 11 items. The content validity index and ratio assessment by 15 experts demonstrated an average (CVI = 0.92, CVR = 0.85).

The classification in the six sections was considered to facilitate its use by midwives who are working in the different fields of care, counseling, education, research, and management. These items are listed in Table 6.

**Table 6:** The final version of the Code of Ethics for midwives in Iran ICEM

Code of Ethics for midwives in Iran ICEM
**1) In the domain of "professional Commitments", the midwife should:**
1–1) know her strengths and weakness regarding the services that she provides;
1–2) increase her competencies by improving her knowledge, skills, attitude, and behavior;
1–3) be conscientious, selfless, and responsible in providing midwifery services;
1–4) know the legal and ethical responsibilities of her profession so as not to harm the quality of providing services;
1–5) provide midwifery services respecting the dignity of clients and respecting their values, religious and cultural beliefs;
1–6) consider the principles of honesty, fairness, politeness, and kindness in providing services;
1–7) takes into account the cultural differences, customs, religion, and language of the clients, and her behavior is far from any kind of discrimination;
1–8) adhere to principles such as keeping the patient's secrets, respecting privacy, respecting individual independence and informed consent;
1–9) consider the principle of non-harm and superiority of the client's interests in providing midwifery services;
1–10) provide midwifery care and services based on professional standards and updated evidence-based information;
1–11) be aware and observe the coordination in the elements of care (including prevention, diagnosis, treatment, and rehabilitation);
1–12) know the existing laws in her different dimensions of work (including educational, research, managerial, and clinical activities) and adhere to them;
1–13) make decisions within the legal, ethical, cultural, and religious frameworks and based on scientific and evidence-based information;
**1–14) Play an effective role in promoting the health of women, families, and society**;
**1–15) Respond to the special needs of vulnerable clients such as high-risk mothers and infants, women with chronic diseases, or AIDS, or alcohol and drug addiction, victims of violence, the homeless, prisoners, refugees, immigrants, and the elderly in providing midwifery services;**
1–16) Providing safe services in a safe environment is a public responsibility;
1–17) provide midwifery services under existing regulations and instructions and report accurately and completely;
1–18) provide care in a proper environment regarding light, sound, heat, cleanliness, order, safety, and cheerfulness;
1–19) be meticulous in maintaining the safety of herself, the clients, and colleagues;
1–20) take necessary precautions to prevent diseases transmitted through blood, **breath,** and fluids;
1–21) provide on-time services with the appropriate follow-up;
1–22) implement the doctor's written orders;
1–23) be responsible for maintaining her professional competence and her behavior does not hurt her profession;
1–24) avoid accepting credits and privileges from the client and her companions, which causes moral problems in the present and future;
**1–25) Be aware of the professional responsibilities of using social media**
**1–26) Provide midwifery services in exceptional circumstances, such as disasters (eg. earthquakes and floods) or an epidemic or pandemic of diseases (eg. the Covid-19 pandemic)**
**2) In the domain of "providing midwifery services to the client and her companions", the midwife should:**
2–1) introduce herself and greet the client and use an identification card that shows her name and professional role;
2–2) establish effective communication with the patient and her companion with kindness and calmness to reduce their anxiety and help meet their needs;
2–3) ask the questions in a simple, clear, and understandable way for the clients and avoid judging her responses;
2–4) consider adequate time to answer the client's questions and educate the client and her companion;
**2–5) Respond to the physical, psychological, spiritual, emotional, cultural, and social needs of the clients who are seeking midwifery care;**
**2–6) Educate self-care to clients who seek midwifery care services**
2–7) provide the necessary information and skills to the client for her participation in the care process;
2–8) develop her knowledge about the culture and language of the area for effective communication with clients and colleagues;
2–9) consider the client's information confidential and do not share it with others without her consent, except in legal cases such as those that threaten her,** her family, or society, and provide the minimum information**
2–10) keep the client's information confidential and endured her that the information would only be shared with the treatment or research team;
**2–11) Protect the client's information in the electronic transfer processes (through information technology, e-mail, fax, and social media applications) by considering the legal aspects**
2–12) provide the necessary explanation before and during the services and inform the client about the results;
2–13) inform the client about her condition in all stages of care and make her and her family feel calm and safe;
2–14) provide a simple and understandable explanation about the benefits and harms of any care services (such as prescribing contraceptives) for making an informed decision;
2–15) explain the reason for any action (such as ultrasound for fetal health assessment);
2–16) obtain permission to transfer sensitive and confidential information to the companions;
2–17) be honest in dealing with the client and her companion;
2–18) be sensitive to human dignity by considering the privacy of the client while providing midwifery services;
2–19) provide midwifery services regardless of the client's sex, age, race, position, economic status, lifestyle, culture, political beliefs, and physical abilities;
2–20) consider the principle of individual independence and get permission from the client before providing any services;
**2–21) Respect the client's autonomy, preferences and beliefs in choosing or rejecting treatment;**
2–22) consider the client's or her guardian's level of knowledge and culture for providing the information, obtaining the consent, and making an informed decision;
2–23) provide midwifery services based on updated information and correct and fair decisions;
2–24) provide midwifery services that benefit the client (such as timely initiation of breastfeeding);
2–25) avoid performing invasive procedures without obstetric indication for the client, such as placing shaving, enema, induction, and episiotomy;
2–26) permitting the client's companion to participate in some care procedures if the client wishes;
2–27) respect the request of the client and his companion to choose a specific person to provide some services;
2–28) respects ​​the supportive role of the client's husband and family in the perinatal period;
2–29) be patient when facing different thoughts, opinions, and behaviors;
2–30) report any case of violation of the client's rights to the relevant official;
2–31) provide the necessary recommendations and training to the patient and her companion before discharge and schedule the follow-ups
**3) In the domain of "relationship with colleagues", the midwife should:**
3–1) know that the atmosphere governing the medical staff located in a department (in terms of compliance with ethical standards) is effective on the clients’ care services;
3–2) collaborate with her colleagues to provide quality midwifery services so that the client's needs can be met;
3–3) communicate with her colleagues respectfully, patiently, supportively, constructively, based on trust, flexible and away from stress and conflict;
3–4) respect the knowledge, experiences, expertise, views, feelings, opinions, priorities, and performance of her colleagues and consider them as valuable resources;
3–5) accept the differences between colleagues, value them and consider the need to have relationships away from discrimination in their profession;
3–6) pay attention and emphasize the role and expertise of other professionals who provide care and support services for clients;
3–7) report any cases of non-compliance with safety, incompetence, immorality, or illegality of services, to the responsible authorities;
3–8) respect the rights of colleagues and members of other disciplines who participate in informed, appropriate and correct decision-making;
3–9) be a positive midwifery model for the students;
3–10) behave respectfully with other midwives, professors, and students (if the center is educational);
3–11) take action by consulting with other colleagues or referring to competent care centers to solve the problem, if there is a problem
**4) In the domain of ​​" herself" the midwife should:**
4–1) try to develop spiritual and moral principles and institutionalize them in herself;
4–2) maintain her dignity and serve with self-respect;
4–3) be aware of her rights in the system and attempts to achieve the **rights through the official legal ways**
4–4) be aware of the rights and rules of the midwifery profession (such as the shifts, the ratio of midwives to clients, working hours, the wage, etc.) and try to solve the challenges in this field;
4–5) be diligent about maintaining her physical-, mental-, social- and spiritual health to provide higher-quality care;
4–6) ensures the confidentiality of her personal information and which is not available to others without the legal standards
**5) In the domain of "education and research", midwives should:**
5–1) considering policy makers’ opinions about the midwifery services that need research and improvement;
5–2) respect the right of people to participate or not participate in any research activity and assure them that their decision will not affect their services;
5–3) try to improve student's knowledge and skills as a teacher and improve their moral and professional practice;
5–4) respect the client's rights and consider ethics when clients are involved in the education;
5–5) be aware and improve her knowledge and ethics in midwifery;
5–6) respect the right of people to participate or not participate in any student’s education process and assure them that their decision will not affect their services;
5–7) not use her professional position to convince the client to participate in the research;
5–8) apply the principles and rules of ethics in research
**6) In the domain of "management", a midwife should:**
6–1) monitor and evaluate the quality of midwifery care processes and structure of services based on the standard;
**6–2) Promote the knowledge and practice of midwifery through research and development of midwifery care standards;**
6–3) ensure specific job descriptions for providing midwifery services in her management part;
6–4) ensure the adequacy, appropriateness, and sterility of the equipment to provide services;
6–5) ensure the safety of the work environment, tools, and equipment;
6–6) review and revise the guidelines, professional and ethical standards, and retraining courses for continuous quality improvement of midwifery services;
6–7) consider adherence to ethical principles in selecting midwifery students and staff;
6–8) ensure adequacy and appropriateness of the facilities for resting the clients' companions;
6–9) attempt to meet the rights of the midwives;
6–10) plan to decrease the stresses caused by the midwifery profession based on the research;
6–11) be familiar with the client's rights in providing services

## Discussion

Considering that all countries have specific regulations and job descriptions in the midwifery service delivery systems, which are based on the cultural, economic, and social conditions of that country [39], the development of a specific code of ethics for every country and even each profession is necessary. Therefore, this study used an innovative approach to develop a valid midwifery code of ethics for the first time in Iran. The items were generated from the content analysis of the interviews with the midwifery and ethics experts who were familiar with providing midwifery services as well as the cultural and social condition, and then the items were completed by reviewing the code of ethics of valid sources in the world and finally, the validity of the content was measured and confirmed by the experts.

Midwives in Iran with bachelor's and master's degrees provide counseling and care services during the perinatal period within the framework of defined tasks in health centers, hospitals, and private offices to women with normal pregnancies. Midwifery graduates with Ph.D. degrees are also employed for teaching and research activities at universities and research centers as well as management jobs. Since Iran has a population with different ethnicities, including Persians, Turk, Kord, Turkmen, Arabs, Gilaks, Lor, Mazan, Baluch, etc., with different religions and cultures, the familiarity of midwives with different customs and beliefs and providing respectful care services and responding to their special needs are essential. In addition, since midwifery services are effective on the life of two humans including the mother, and the fetus or infant providing quality respectful services while considering ethical issues is critical [40].

The finding of the qualitative phase of the study defined midwifery ethics as "considering ethical issues in providing care for the client, in interactions with her companions, about midwife herself, and about the environment and procedure of the services”. Regarding the wide range of midwifery tasks in providing services to the client, many items were included in the category "considering ethical issues in providing care for the client”. The items of this dimension generally refer to the client's rights, empathy, keeping privacy and confidentiality, as well as paying attention to their autonomy, dignity, desires, and needs, reducing stress, and providing respectful and safe quality care without any discrimination. In recent years, the promotion of providing care based on professional ethics and dignity has attracted the attention of countries and international organizations, and respectful maternity care is considered an essential concept for ensuring the rights and safety of women during labor [35]. The important ethical issues about clients are commonly mentioned; avoiding harm and maltreatment, having the right to get information, having the right to informed consent or refuse, respect for the patient's choices and preferences, privacy and confidentiality of information, considering the clients’ dignity and providing timely and quality care without any discrimination [16]. Considering ethical issues and providing respectful maternal care through polite and supportive communication between the woman and the midwife increases the client's self-confidence and provides a basis for respecting the client's rights [41].

The midwifery ethical items about the relationship between midwives and the client's companions showed two main categories including giving the relevant information and permitting them to participate in the care procedures. Clients' companions are mostly the family who are concerned about the client's health and try to get information about the client’s condition [42]. Family-centered midwifery care is a critical model in midwifery and maternal care [43, 44]. In this model of care, it is important to make proper communication with clients and their families, understand their concerns, and answer their questions [45]. Besides, understanding their needs is effective in the communication process causes mutual trust, and improves respectful and ethical care procedures [41].

The midwifery ethical items about colleagues showed the importance of considering respect, protection, proper reporting, and cooperation with other midwives and the team members. Cooperation in team working is an effective interpersonal skill that facilitates achievement the of goals, and quality care cannot be achieved when the care providers act alone [46]. Effective communication between colleagues improves health outcomes as they can share their experiences for providing quality care service [44].

Attention to the rights of midwives as well as training retraining and self-monitoring were mentioned as the most important ethical considerations about the midwife herself. Midwives have a key role in improving maternal-fetal and neonatal health as well as women's sexual reproductive health. Therefore, midwives’ continuous education is necessary to improve maternal and women’s health and the quality of care [47]. Education is a basic element for increasing professional midwives, improving professional and evidence-based practices under international standards, and guaranteeing high-quality services [1]. Developing standards such as the code of ethics for midwives and other health personnel, with cultural consideration can also help the health providers self-monitoring their practice [48] and then improve the quality of care [49].

The ethical considerations about the work environment including the physical environment as well as the facilities and equipment were also stated as an important aspect of the ICEM. No doubt providing quality care with ethical considerations requires an appropriate environment with adequate and efficient equipment and facilities that lead to clients’ satisfaction [50]. However, providing the necessary environment and facilities requires support at the management level.

Considering ethical issues in the care processes as well as management and administrative processes were mentioned as the main aspects of providing midwifery care services. Providing quality care procedures needs appropriate facilities and equipment that in turn needs financial support which is a prerequisite for providing ethical and standard respectful care and maintaining the dignity of the client [51]. The care procedures must be developed based on the community needs and the structure of the health system of every country, and a universal package cannot be used for all countries because some elements such as resources, management, and capacity of the health system are effective in this process [52].

After extracting the items from the qualitative phase of the study, the items were completed with a review of the code of ethics for midwives of the International Midwifery Confederation ICM, American College of Nursing and Midwifery ACNM, UK Nursing and Midwifery Council UKNMC, The British Columbia College of Nurses and Midwives BCCNM, Nursing and Midwifery Board Australia NMBA, New Zealand College of Midwives NZCM, the national code of ethics in Turkey, and Nursing and Midwifery Board of Irland [31–38]. The finding showed that all the above-mentioned codes of ethics as well as the ICEM are developed based on the four principles of medical ethics including respect for autonomy, non-maleficence, beneficence, and justice. In addition, all the documents have similar items but with different specifications and details. It seems ICEM is more similar to the UK Nursing and Midwifery Council which has 95 items and similar details. Besides our findings the review highlighted some items related to recent social-, scientific, and technical improvements such as; 1) responding to the client’s needs in exceptional conditions like disasters and epidemic or pandemic diseases; 2) The social role of midwives in promoting sexual-reproductive health in the community and self-care and the use of media; 3) confidentiality and privacy of client's information while transferring information through internet and, 4) considering all physical, psychological, and social health needs of the clients.

As it is mentioned above, “responding to the client’s needs in exceptional conditions like disasters and epidemic or pandemic diseases”, was an important added item to the IMEC. In such a condition, insufficient access to medical and health care services and food, and an increase in STIs usually occur [52, 53]. Some people may experience the loss of a fetus, infant, spouse, or other relatives [54]. Therefore, on-time care and treatment are critical in disasters [55], and midwives need to provide care in crisis such as the necessary special maternal care that was shown recently during the COVID-19 pandemic condition [56, 57].

One other added important code was “The social role of midwives to promote sexual-reproductive health in the community and self-care and the use of media”. The presence of midwives in social networks causes them to be seen and popularized in society [58]. Midwives can play a social role and use social media to provide evidence-based information for promoting sexual-reproductive health behaviors in the community, family, and especially among women. This is ethically a main responsibility of midwives [59].

Since midwives have access to the most private information of people, confidentiality in the recent techniques for internet transfer of the information is so important. Although information transfer through the Internet makes easy and quick access to the client's information in the health system, monitoring the confidentiality of the information is critical [59].

For the first time, we used a mixed sequential study to develop the ICEM, in three phases including a qualitative study, a review of the codes of ethics for midwives in the world, and then a qualitative and quantitative assessment of content validity of the items. Fifteen experienced and skilled experts reviewed the content of the items and presented their opinions for the necessary improvement, and the necessary corrections were performed. Then the experts scored the essentiality and relevancy of the items. The calculated average CVI and CVR were calculated and confirmed by an average of 0.92 and 0.85, respectively. This version of ICEM has 92 items compared to the previous version which had 85 items. It contained 7 further items in terms of keeping confidential in transferring the client's information via the Internet; responding to the client's needs in special circumstances such as epidemics and disasters, and responding to the needs of different vulnerable clients; paying attention to all aspects of clients’ health, have the responsibility in playing social roles in promoting maternal and women's health and also self-care in the community.

Based on the opinion of the experts for making the ICEM easier for use by different groups of midwives working in clinical, educational, research, and management, the ICEM was developed in 6 domains including; "professional commitments", "providing midwifery services to the woman and her companions", "relationship with colleagues", "herself", "education and research" and "management". To do this, the categories of “client and the client accompanies” in the qualitative phase were integrated to form the "the woman and her companions" section in ICEM. Also the categories of “procedures and environment” were merged and formed the “management” section of ICEM. The items related to the common responsibilities of all midwives merged in the "professional commitment" section. The items related to education and research were also merged in the "education and research" section to be used for midwifery educators and researchers.

Finally, it should be noted that to our knowledge this was the first time that a mixed sequential method was used for developing a professional code of ethics. Although, Kangasniemi and colleagues developed an ethic guideline for nurses’ collegiality using the 4-step Delphi method [60]. Therefore, using this method as an innovation could be considered as the strength of the study.

We refer to “her” or “herself” throughout the items of ICEM because all midwives are female in Iran. As the ethical code could be used internationally, we recommend to use with gender-neutral pronouns. The AGREE-HS (Appraisal of Guidelines Research and Evaluation Health Systems) is a newly developed tool designed to evaluate the quality of health systems guidance (HSG) documents and provides a blueprint for HSG document development and reporting [61].

## Conclusion

We used a scientific process to prepare a valid ICEM. The classification in the six sections including "professional Commitments”, "providing midwifery services to the woman and her companions", "relationship with colleagues", "herself", "education and research" and "management" were considered to facilitate its use by midwives who are working in the different fields of care, counseling, education, research, and management. In this new version of the ICEM, the items related to recent social-, scientific, and technical improvements such as responding to the client's needs in exceptional conditions like disasters and epidemic or pandemic diseases; the social role of midwives in promoting sexual-reproductive health in the community and the use of media; confidentiality of client’s information while transferring information through the internet were also considered.

### Supplementary Information

**Additional file 1.**

## Data Availability

All relevant raw data will be freely available to any scientist wishing to use them for non-commercial purposes, without breaching participant confidentiality. The datasets generated and/or analyzed during the current study are not publicly available because sending the data needs obtaining permission from the university but are available from the corresponding author (Masoumeh Simbar) upon reasonable request.

## Abbreviations

ICEM: Code of Ethics for midwives in Iran
CVR: Content Validity Ratio
CVI: Content Validity Index
ACNM: The American College of Nurses and Midwives
ICM: International Confederation of Midwives
MANA: The Midwifery Association of North American
NZCM: The New Zealand College of Midwives
BCCNM: The British Columbia College of Nurses and Midwives
NMBA: The Nursing and Midwifery Board Australia
UKNMC: The UK Nursing and Midwifery Council
AIDS: Acquired Immune Deficiency Syndrome
AGREE-HS: Appraisal of Guidelines Research and Evaluation Health Systems
HSG: Health Systems Guidance
