# Supplementary material for: Development and validation of the code of ethics for midwives in Iran
Source: BMC Med Ethics. 2023 Oct 4;24:76. doi: 10.1186/s12910-023-00963-4 (PMC10548606; doi:10.1186/s12910-023-00963-4)
Supplement: Supplementary file 1 — Additional file 1. [file 12910_2023_963_MOESM1_ESM.docx]

**Supplement 1**

**The interview guide for the study “Development and validation of the Code of Ethics for Midwives in Iran”**

"What is the concept of midwifery ethics?"

"What could be the dimensions of this concept?"

"How midwifery ethics could be considered in midwifery services"

“How can you interpret these dimensions in different aspects of midwifery practice such as care, education research and management”

The probing questions such as "How?", “What do you mean by that?" and “Please elaborate on this point” were also used.
